# The *Epichloë festucae* antifungal protein has activity against the plant pathogen *Sclerotinia homoeocarpa*, the causal agent of dollar spot disease

**DOI:** 10.1038/s41598-017-06068-4

**Published:** 2017-07-17

**Authors:** Zipeng Tian, Ruying Wang, Karen V. Ambrose, Bruce B. Clarke, Faith C. Belanger

**Affiliations:** 1 Department of Plant Biology, Rutgers University, New Brunswick, New Jersey 08901 USA; 2 Indigo Agriculture, Charlestown, Massachusetts 02129 USA

## Abstract

*Epichloë* spp. are naturally occurring fungal endophytic symbionts of many cool-season grasses. Infection by the fungal endophytes often confers biotic and abiotic stress tolerance to their hosts. Endophyte-mediated disease resistance is well-established in the fine fescue grass *Festuca rubra* subsp. *rubra* (strong creeping red fescue) infected with *E*. *festucae*. Resistance to fungal pathogens is not an established effect of endophyte infection of other grass species, and may therefore be unique to the fine fescues. The underlying mechanism of the disease resistance is unknown. *E*. *festucae* produces a secreted antifungal protein that is highly expressed in the infected plant tissues and may therefore be involved in the disease resistance. Most *Epichloë* spp. do not have a gene for a similar antifungal protein. Here we report the characterization of the *E*. *festucae* antifungal protein, designated *Efe*-AfpA. The antifungal protein partially purified from the apoplastic proteins of endophyte-infected plant tissue and the recombinant protein expressed in the yeast *Pichia pastoris* was found to have activity against the important plant pathogen *Sclerotinia homoeocarpa*. *Efe*-AfpA may therefore be a component of the disease resistance seen in endophyte-infected strong creeping red fescue.

## Introduction

*Epichloë* spp. are common naturally occurring fungal endophytic symbionts of many cool-season grasses^1, 2^. The fungi are systemic within the above-ground portions of the plant. They grow exclusively in between the plant cells through a novel intercalary hyphal extension process^3^. Infection by the fungal endophytes often confers biotic and abiotic stress tolerance to their hosts^4^. Resistance to insect and mammalian herbivory has been associated with the synthesis of toxic alkaloids by the fungal endophytes^5^. In some endophyte-plant associations, drought tolerance and disease resistance have also been attributed to infection by fungal endophytes, but the underlying mechanisms are not fully understood^6–9^.

The fine fescues strong creeping red fescue (*Festuca rubra* subsp. *rubra*) and Chewings fescue (*Festuca rubra* subsp. *commutata*) are commercially important low-maintenance turfgrasses^10^. The main location of seed production in the United States is Oregon. Over 18 million pounds of *Festuca rubra* seed were produced in Oregon in 2015 (http://cropandsoil.oregonstate.edu/system/files/u1473/GrassSeedProduction2015.pdf).

Endophyte-infected cultivars are generally preferred because of their enhanced insect, stress, and disease resistance. Endophyte-mediated disease resistance to the fungal pathogens *Laetisaria fuciformis* (McAlp.) Burdsall, the causal agent of red thread disease, and *Sclerotinia homoeocarpa* Bennett, the causal agent of dollar spot disease, has been well established in the fine fescues^6, 7^. Both diseases are serious management problems for *Festuca rubra* ^7, 11^. Current breeding efforts include incorporation of *E*. *festucae* into new cultivars to reduce the necessity for fungicide use for disease control^7, 12^.

Disease resistance as observed in the endophyte-infected fine fescues is not a general feature of endophyte-infected grasses^13^. No such endophyte-mediated *Sclerotinia homoeocarpa* resistance was seen in perennial ryegrass (*Lolium perenne* L.) field trials^7^. Similarly, no endophyte-mediated resistance to brown patch caused by *Rhizoctonia solani* Kuhn or to *Puccinia graminis* subsp. *graminicola* was observed in tall fescue (*Schedonorus arundinaceus* (Schreb.) Dumont. = *Lolium arundinaceum* (Schreb.) Darbysh., formerly *Festuca arundinacea* Schreb.)^14, 15^. Overall, breeders have not reported endophyte-mediated resistance to fungal pathogens in the cultivated grasses perennial ryegrass or tall fescue.

The literature on effects of *Epichloë* endophyte infection on disease resistance of various host grasses is conflicting. Hume *et al*.^16^ present a comprehensive summary of some of the conflicting results. Endophyte-mediated disease resistance may therefore be unique to the fine fescues since field-level disease resistance has not been conclusively established in other *Epichloë*-grass associations.

In a quantitative transcriptome study of the *Epichloë festucae*-strong creeping red fescue interaction^17^, the second most abundant fungal transcript, representing 6% of the fungal transcripts *in planta*, encoded a protein similar to antifungal proteins from *Penicillium* and *Aspergillus* species^18^. Based on the activity assays described below for the *E*. *festucae* protein, the designations of similar genes from other fungal species, and nomenclature recommendations for *Epichloë* spp.^19^, the *E*. *festucae* gene is designated *Efe*-*afpA* and the protein is designated *Efe*-AfpA. The antifungal protein gene found in *E*. *festucae* infecting strong creeping red fescue is not present in most *Epichloë* genomes for which whole genome sequence is available^17^. The transcript abundance and the limited existence of the gene among *Epichloë* spp. suggested the *E*. *festucae* antifungal protein may be a component of the unique endophyte-mediated disease resistance observed in strong creeping red fescue. Here we report the functional characterization of the *E*. *festucae* antifungal protein. The purified recombinant antifungal protein was found to have activity against the important turfgrass fungal pathogen *Sclerotinia homoeocarpa*, the causal agent of dollar spot disease.

## Results

### The *E*. *festucae* Antifungal Protein is a Component of the Apoplastic Proteins from Infected Strong Creeping Red Fescue

**Figure 1:**
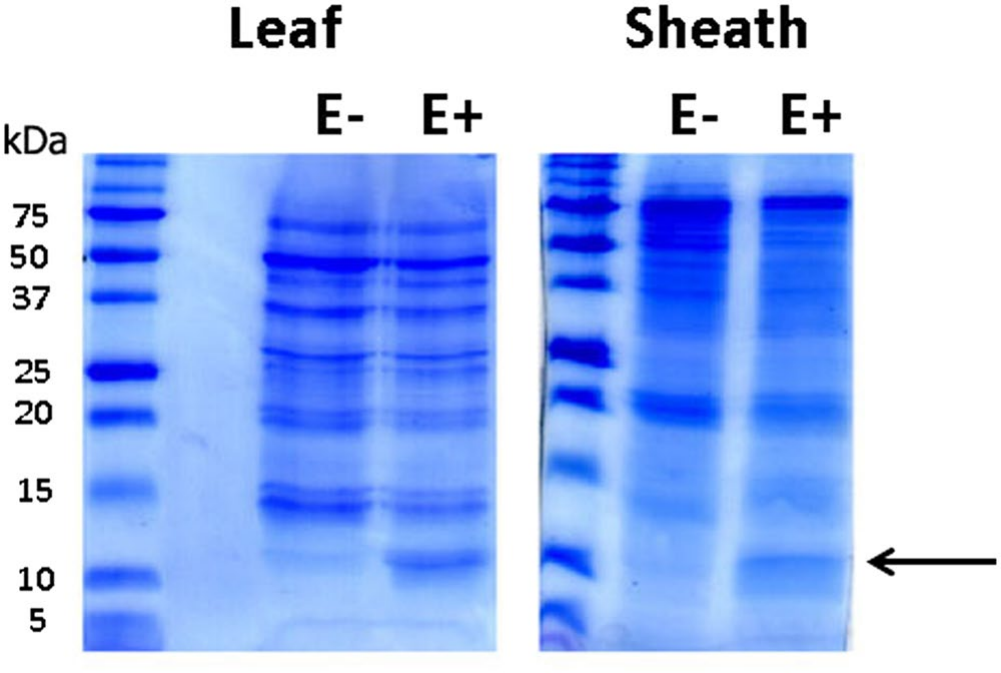
SDS-PAGE analysis of apoplastic proteins from leaf and leaf sheath tissue of endophyte-free (E−) and endophyte-infected (E+) plants. Protein standards molecular masses in kDa are indicated on the left side of the figure. The arrow indicates the band cut for protein sequence analysis.

The *E*. *festucae* antifungal protein *Efe*-AfpA (gene model EfM3.063660) was predicted to be a secreted protein by the program TargetP^17, 20^. As a secreted protein, it was expected to be in the apoplastic fluid of the infected plant tissue. Therefore the apoplastic proteins were isolated from leaves and leaf sheaths from endophyte-free and endophyte-infected plants and compared on an SDS-polyacrylamide gel (Fig. 1). A band at about 10 kDa was more prominent in both the leaf and leaf sheath samples from the endophyte-infected plant than in the samples from the endophyte-free plant.

The 10 kDa band was excised from the endophyte-infected leaf sheath sample and subjected to LC/MS/MS peptide sequencing. The *E*. *festucae* antifungal protein was the most abundant protein detected in the sample, confirming that it is indeed a secreted protein. Numerous other *E*. *festucae* proteins were also detected at lower abundance and information on those proteins is presented in Supplementary Table S1. No plant proteins were detected, likely because there is limited sequence information available for the host, strong creeping red fescue.

### Protein Sequence Comparisons of the *E*. *festucae* Antifungal Protein with Similar Antifungal Proteins

**Figure 2:**
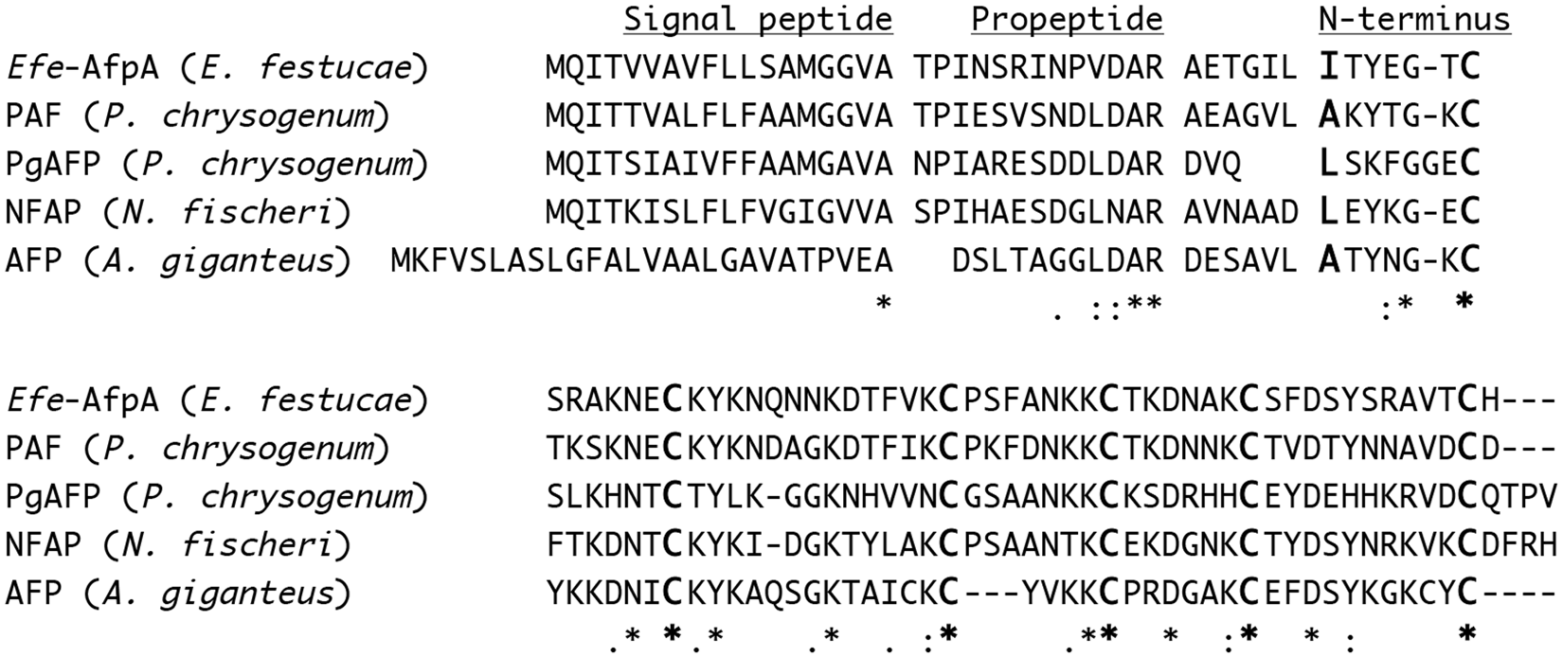
Amino acid alignment of *Efe*-AfpA from *E*. *festucae* (accession SRR493691.12929) with PAF from *P*. *chrysogenum* (accession U22944.2), PgAFP from *P*. *chrysogenum* (accession D0EXD3.1), NFAP from *N*. *fischeri* (accession AM983570), and AFP from *A*. *giganteus* (accession X60771). The experimentally determined N-terminus of each protein is in bold larger font size. The conserved cysteines are also in bold larger font size. Breaks in the sequences were introduced to indicate the predicted signal peptide cleavage sites, the proposed propeptide cleavage sites, and the proposed final processing sites. An asterisk indicates identical residues in all sequences, a “:” indicates strongly conserved residues (score >0.5), and a “.” indicates weaker conserved residues (score <0.5)^65^.

The *E*. *festucae* antifungal protein sequence is similar to proteins from several other fungal species from the subphylum Pezizomycotina^17^. The most well-characterized are designated AFP (antifungal protein) from *Aspergillus giganteus* ^21, 22^, NFAFP from *Neosartorya fischeri* (synonym *Aspergillus fischeri*)^23^ and two similar yet distinct proteins, PAF and PgAFP, from *Penicillium chrysogenum* ^24–26^. A comparison of these protein sequences with the *Efe*-AfpA sequence is shown in Fig. 2. Phylogenetic analysis of the amino acid sequences indicates the *Efe*-AfpA sequence is more similar to PAF than to the other antifungal protein sequences in the comparison (Supplementary Fig. S1), as was reported previously for the DNA coding sequences^17^. All of these characterized proteins are known to be secreted and the predicted signal peptide cleavage sites are indicated in Fig. 2. The experimentally determined N-termini of the similar proteins are indicated in Fig. 2. In all cases it is 16 to 19 amino acids downstream of the signal peptide cleavage site, indicating there is additional processing of these proteins^21, 23, 26, 27^.

The most N-terminal peptide detected from *Efe*-AfpA was AETGILITYEGTCSR (TIC 6.3e5). However, the peptide ITYEGTCSR was considerably more abundant, with a TIC of 3.3e7, suggesting it was the N-terminus of the predominant form of the protein. The *A*. *giganteus* AFP was reported to exist in 2 forms, with the N-terminus of the predominant form being the alanine indicated in Fig. 2 ^28^. However, on short culture times of 48–60 h, a longer minor component with 6 additional amino acids at the N-terminus was detected in addition to the major form. Similarly, the *Efe*-AfpA minor form also had an additional 6 amino acids at the N-terminus. Based on a comparison of fifteen secreted proteins from *Aspergillus* spp., Martinez-Ruiz *et al*.^28^ identified the common tetrapeptide LDAR at the C-terminus of the propeptides of those secreted proteins. A similar sequence is found in all the antifungal proteins shown in Fig. 2. This sequence does not conform to the canonical dibasic kexin cleavage site of (K/R)R and is not recognized by the program ProP designed to detected propeptide cleavage sites^29^. However, the propeptides of some fungal secreted proteins are cleaved by yapsins, which are aspartic peptidases that can cleave C-terminal to both mono- and dibasic residues^30^. If the LDAR tetrapeptide is indeed the C-terminus of the *E*. *festucae* and *A*. *giganteus* antifungal protein propeptides, that would suggest that the other antifungal proteins may also be secreted as longer forms that subsequently are proteolytically cleaved to the mature form. However, such longer forms have only been identified in *A*. *giganteus* and now in *E*. *festucae*. The longer form of the *A*. *giganteus* AFP was considerably less active than the mature form^28^, as was the longer form of *Efe*-AfpA (described below).

The mature form of *Efe*-AfpA has 55 amino acids with a molecular weight of 6,278 Daltons, and a pI of 8.9. The difference in the calculated molecular weight of the mature *Efe*-AfpA of approximately 6.3 kDa and its migration in SDS-PAGE at approximately 10 kDa is similar to that reported for PgAFP, which was attributed to the extreme pI of the protein^26^. NMR solution structures have been determined for AFP and PAF and both proteins have compact structures held together with disulfide bonds in the hydrophobic interior core of the protein, although the precise cysteine pairing in the disulfide bonds could not be determined^31, 32^. The cysteine pairing in PAF was determined by mass spectrometry of disulfide-bonded peptides^33^. The cysteine residues in PAF are conserved in *Efe*-AfpA (Fig. 2). Based on the conserved cysteines, as well as the overall sequence similarity, it seems likely that the tertiary structure of *Efe*-AfpA is similar to that of PAF.

### Antifungal Activity of *Efe*-AfpA Partially Purified from Apoplastic Proteins

**Figure 3:**
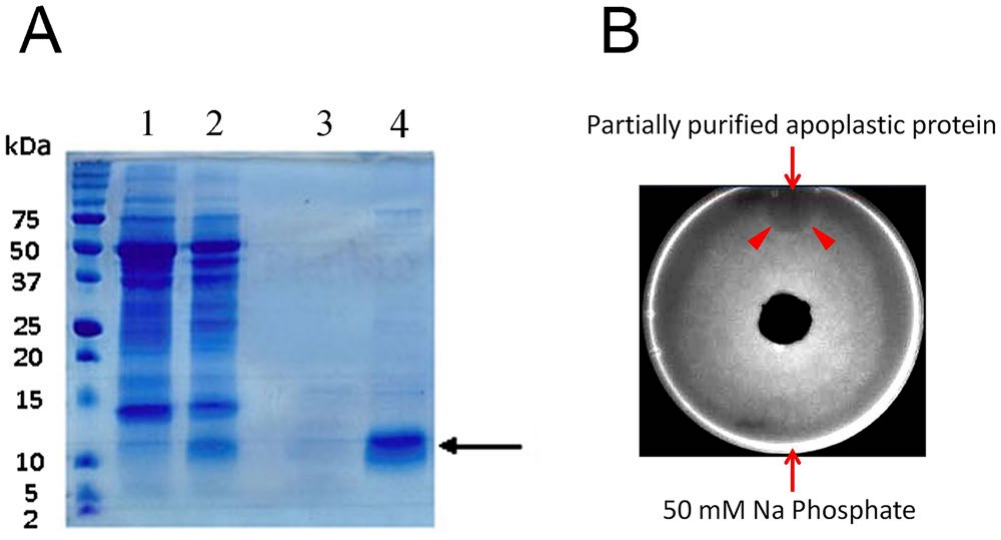
Partial purification of *Efe*-AfpA from apoplastic proteins and its activity against the dollar spot fungus. (**A**) SDS-PAGE analysis of partially purified *Efe*-AfpA from apoplastic proteins. Lane 1, total apoplastic proteins from leaves of endophyte-free plants; Lane 2, total apoplastic proteins from leaves of endophyte-infected plants; Lane 3, unbound fraction from the HiTrap phenyl HP column; Lane 4, desalted proteins eluted from the HiTrap phenyl HP column with 50 mM NaPO_4_, 1 M (NH_4_)_2_SO_4_ (pH 7.0). Arrow indicates band cut for protein sequencing. (**B**) Plate assay of antifungal activity of the partially purified *Efe*-AfpA from apoplastic proteins against the dollar spot fungus. A plug of the dollar spot fungus was placed in the center of the plate and 30 μL (1 μg μL^−1^) of *Efe*-AfpA and 50 mM NaPO_4_ were spotted at opposite ends of the plate. Arrow indicates region of growth inhibition at position of *Efe*-AfpA application.

*Efe*-AfpA was partially purified from the crude apoplastic proteins of endophyte-infected plants through hydrophobic interaction chromatography (Fig. 3A). The prominent band at about 10 kDa in the partially purified sample was confirmed to be *Efe*-AfpA by protein sequencing. The partially purified *Efe*-AfpA had activity against the *Sclerotinia homoeocarpa* fungus in a plate assay as seen by a zone of no growth of the fungus at the site of *Efe*-AfpA application (Fig. 3B).

### Expression of Efe-*afpA* in *Pichia pastoris*

Since the antifungal activity of *Efe*-AfpA isolated from apoplastic proteins was confirmed, it was expressed in the yeast *Pichia pastoris* in order to obtain larger quantities of protein for functional characterization. The similar antifungal proteins from *P*. *chrysogenum* (PAF), *A*. *giganteus* (AFP), and *N*. *fischeri* (NFAP) have been successfully expressed in *P*. *pastoris* ^32, 34, 35^. Coding sequences of the long form (designated *Efe*-*afpA*-2) and the major mature form (designated *Efe*-*afpA*-3) of *Efe*-AfpA were cloned into the *P*. *pastoris* expression vector pPICZα A, a vector designed to target the recombinant protein for secretion from the yeast cells. *P*. *pastoris* does not secrete high levels of endogenous proteins, so the expressed recombinant protein is generally the major protein in the culture filtrate.

**Figure 4:**
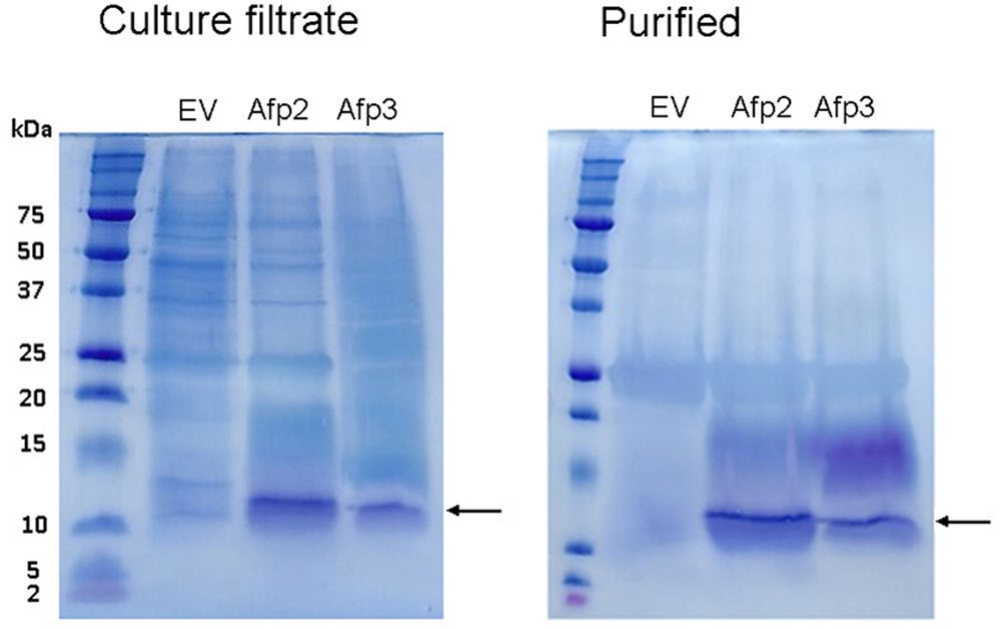
Expression of the *Efe*-*afpA*-2 and *Efe*-*afpA*-3 constructs in *P*. *pastoris*. SDS-PAGE of proteins in the methanol-induced *P*. *pastoris* culture filtrates and the partially purified *Efe*-AfpA-2 and *Efe*-AfpA-3 proteins. Arrows indicates *Efe*-AfpA proteins. EV, proteins from the empty vector control transformant; Afp2, proteins from the *Efe*-*afpA*-2 longer form construct; Afp3, proteins from the *Efe*-*afp*A-3 mature form construct.

Both recombinant forms of *Efe*-AfpA were indeed the major proteins recovered in the culture filtrates concentrated through a 10 kDa molecular weight cutoff centrifugal filter (Fig. 4). *Efe*-AfpA-2 and *Efe*-AfpA-3 were partially purified from the culture filtrate by first passing through a 30 kDa molecular weight cutoff centrifugal filter and then concentrating the flow-through with a 10 kDa molecular weight cutoff centrifugal filter (Fig. 4). Peptide sequencing of the major band at the expected size and the diffuse band at about 15 kDa from the partially purified proteins revealed both were *Efe*-AfpA. A similar diffuse higher molecular weight band was seen in the purification of AFP from *A*. *giganteus* and was attributed to a dimer with rearranged disulfide bonds^28^. The protein band at about 25 kDa seen in the partially purified samples from the empty vector as well as the *Efe*-*afpA* constructs was sequenced and identified as triosephosphate isomerase, whose predicted molecular weight is 27 kDa. Triosephosphate isomerase would not be expected to be secreted and is likely found in the culture filtrate due to some cell lysis, as has been reported previously for methanol-induced cultures of *P*. *pastoris* ^36^.

### Antifungal Activity of *Efe*-AfpA Expressed in *Pichia pastoris*

*Efe*-AfpA-2 and *Efe*-AfpA-3 purified from *P*. *pastoris* culture filtrates as described above were tested for antifungal activity against *S*. *homoeocarpa* in two different assays. Proteins purified in the same way from the culture filtrate of the empty vector transformant were used as controls. Since *S*. *homoeocarpa* does not produce spores, all assays were performed on *S*. *homoeocarpa* mycelium maintained on PDA.

**Figure 5:**
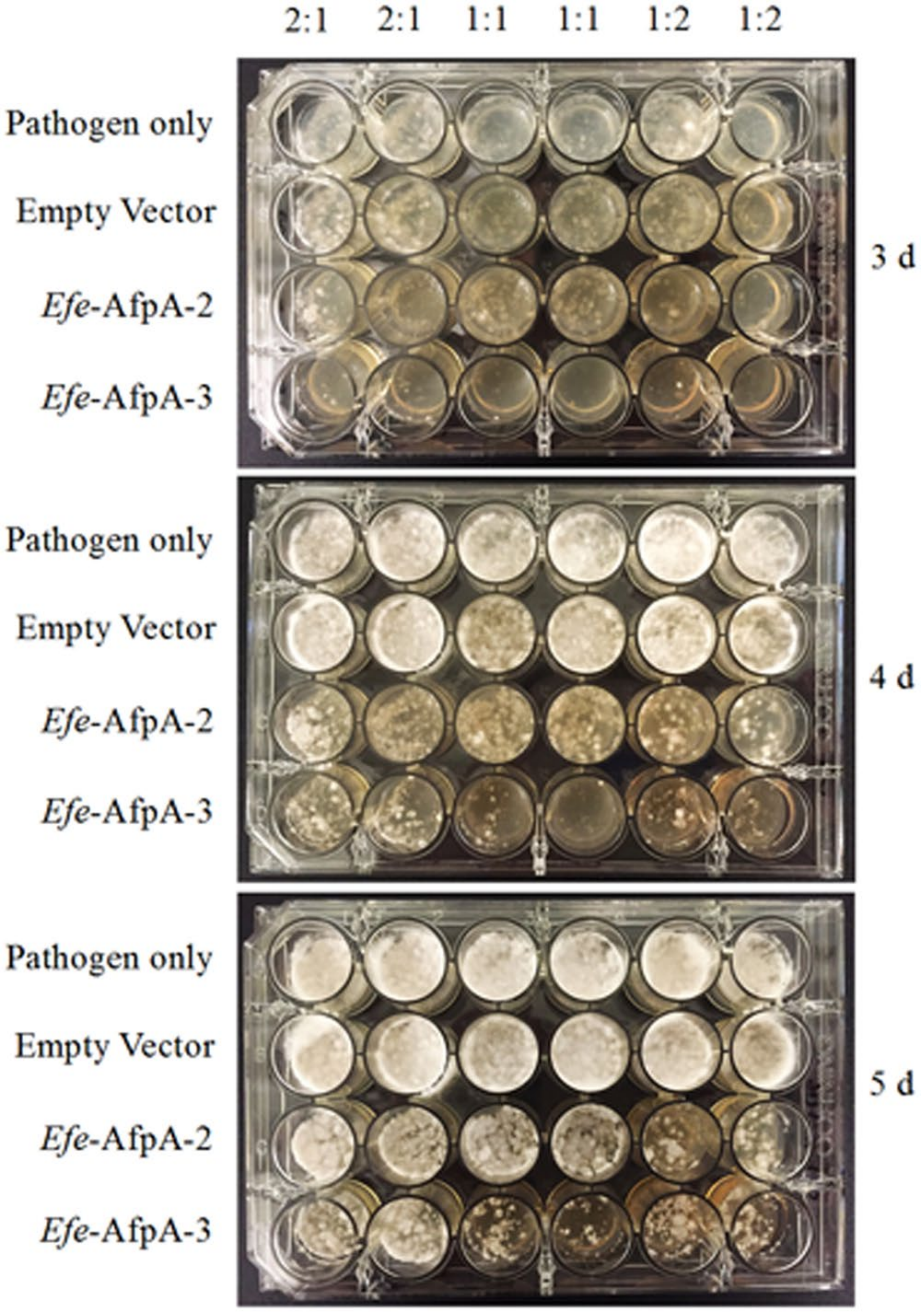
Antifungal activity assay of *Efe*-AfpA-2 and *Efe*-AfpA-3 purified from *P*. *pastoris* culture filtrates against *S*. *homoeocarpa* 3–5 days after inoculation. Different volumes of a *S*. *homoeocarpa* slurry were mixed with the purified proteins in 2:1, 1:1, and 1:2 ratios and plated onto PDA in a 24 well plate. The amounts of purified protein in the mixture are 30 μg, 45 μg, and 60 μg, respectively.

In a qualitative assay for antifungal activity, *S*. *homoeocarpa* mycelium was gently homogenized to a slurry, mixed with *Efe*-AfpA-2 and *Efe*-AfpA-3 in different volume to volume (v:v) ratios, and the mixture applied to PDA in a 24-well plate (Fig. 5). Inhibition of growth of *S*. *homoeocarpa* by both forms of *Efe*-AfpA could be seen by 3 days after plating. The empty vector proteins had no effect on the growth of the pathogen. By 4 days after plating the pathogen only and empty vector samples had completely filled the wells whereas inhibition of growth was apparent for both *Efe*-AfpA forms at all v:v ratios. *Efe*-AfpA-3 resulted in the greatest inhibition of the pathogen.

**Figure 6:**
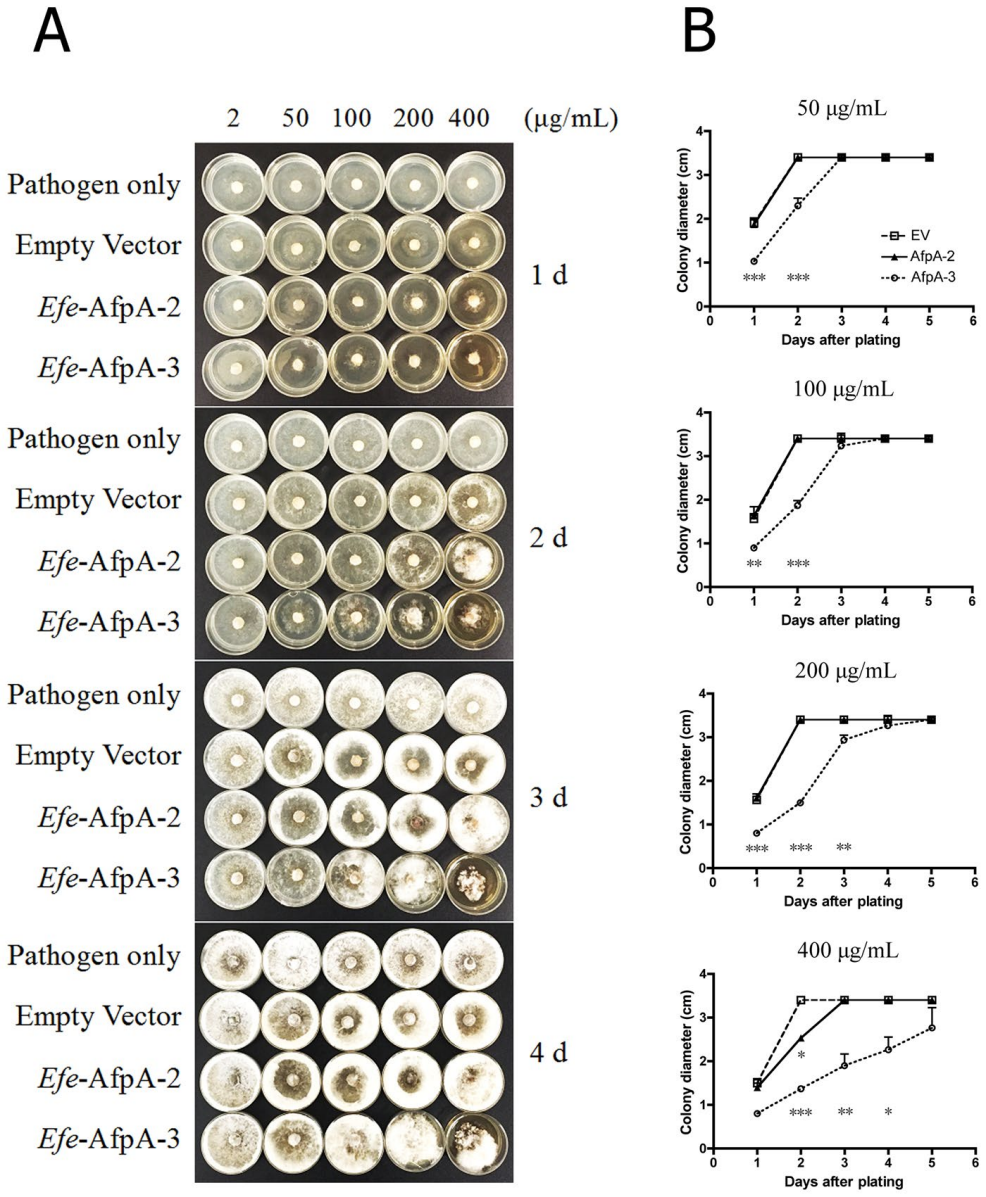
Antifungal activity assay of *Efe*-AfpA-2 and *Efe*-AfpA-3 purified from *P*. *pastoris* culture filtrates against *S*. *homoeocarpa*. The purified proteins, 2–400 μg mL^−1^ were incorporated into 35 mm PDA plates and 0.5 cm plugs of *S*. *homoeocarpa* were placed in the center of the plates. (**A**) Photographs of 35 mm plates 1–4 days after inoculation with *S*. *homoeocarpa*. (**B**) Colony diameter changes of *S*. *homoeocarpa* on the 35-mm plate 1–5 days after inoculation. Significant differences between constructs *Efe*-AfpA-2 and *Efe*-AfpA-3, each relative to the empty vector proteins, are indicated by ***(*P* < 0.001), **(P < 0.01), and *(*P* < 0.05). Vertical bars are standard deviation values for diameter comparisons of each construct at a given day after inoculation.

In a quantitative assay for antifungal activity, different concentrations of *Efe*-AfpA-2 and *Efe*-AfpA-3 were incorporated into 35 mm agar plates and 0.5 cm plugs of *S*. *homoeocarpa* were placed in the center of the plate. Representative plates of the triplicate samples are shown in Fig. 6. With *Efe*-AfpA-2 only the highest concentration, 400 μg mL^−1^, resulted in inhibition of growth of *S*. *homoeocarpa*, whereas *Efe*-AfpA-3 resulted in inhibition at all concentrations except 2 μg mL^−1^.

### *Efe*-AfpA permeabilizes the cell membranes of *S*. *homoeocarpa*

**Figure 7:**
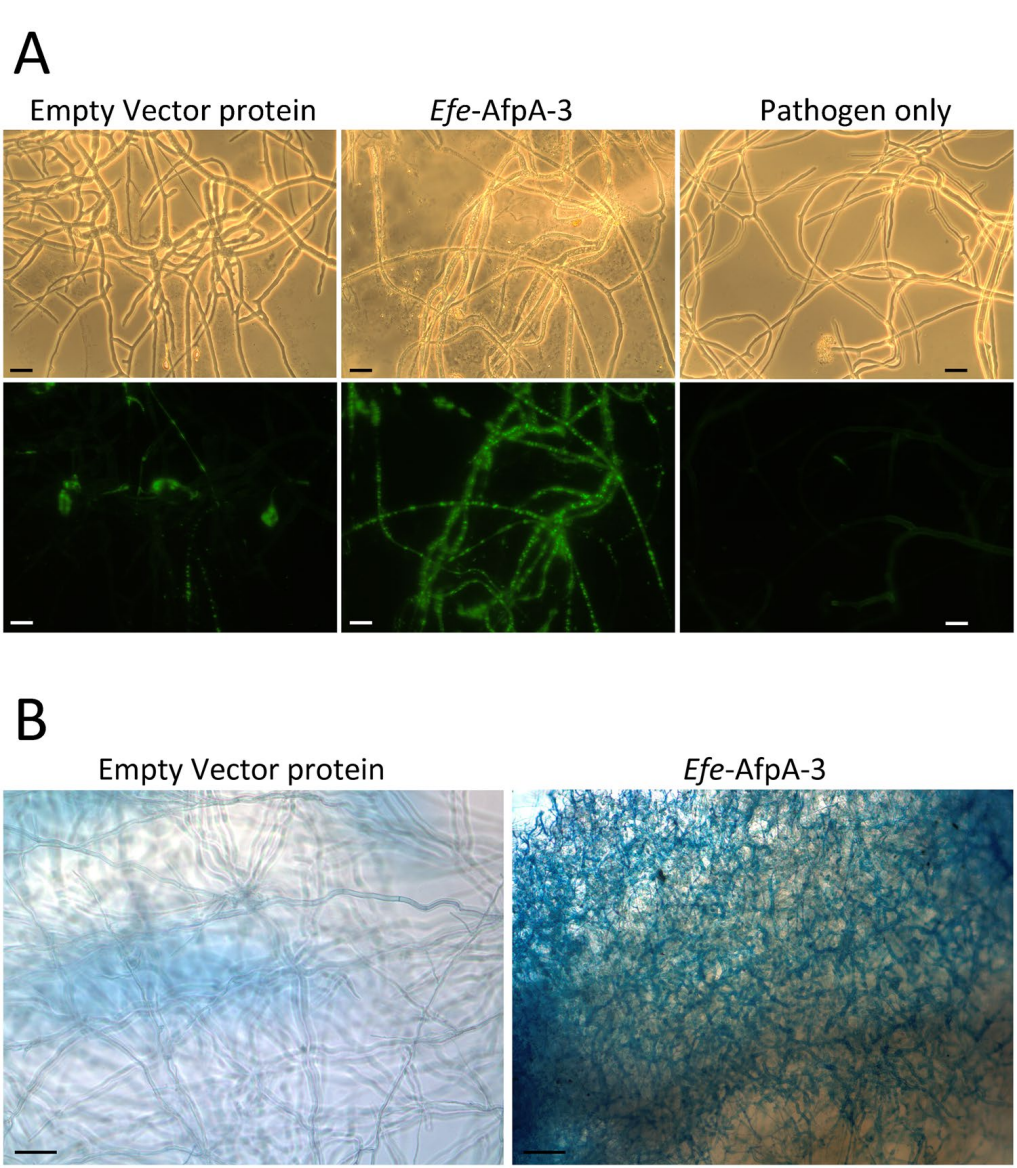
Microscopy of *S*. *homoeocarpa* mycelium treated with *Efe*-AfpA-3 and empty vector proteins purified from the *P*. *pastoris* culture filtrates. (**A**) Detection of SYTOX Green uptake by fluorescence microscopy. *S*. *homoeocarpa* was cultivated in PDB for 2 d, and 17 μg of *Efe*-AfpA-3 or empty vector proteins were added. After 2 h of incubation, fluorescence microscopy was examined in the presence of 12 μM SYTOX Green. The upper panels are bright-field images and the lower panels are fluorescence images of the same fields. The scale bar is 20 μm. (**B**) Detection of Evans blue uptake by light microscopy. *S*. *homoeocarpa* in the center of a PDA coated microscope slide was treated with *Efe*-AfpA-3 or empty vector proteins for 1 d and then stained with Evans blue. The scale bar is 100 μm.

The mechanism of the observed inhibition of growth of *S*. *homoeocarpa* by *Efe*-AfpA was investigated by using the viability stains SYTOX Green and Evans blue. SYTOX Green is a fluorescent nucleic acid stain that can only penetrate cells with compromised plasma membranes, and will not penetrate live cells. Evans blue is a non-fluorescent azo dye that also can only enter cells with damaged plasma membranes. The results of these viability assays are shown in Fig. 7. After 2 h of incubation with *Efe*-AfpA-3, the *S*. *homoeocarpa* mycelia exhibited intense fluorescence of SYTOX Green, whereas mycelia treated with the empty vector proteins or untreated mycelia only showed a low amount of fluorescence (Fig. 7A). Similarly, treatment of *S*. *homoeocarpa* with *Efe*-AfpA-3 resulted in uptake of Evans blue whereas treatment with the empty vector proteins had no effect (Fig. 7B).

### The *E*. *festucae* Antifungal Protein Gene Is Not Present in Most *Epichloë* Genomes

**Figure 8:**
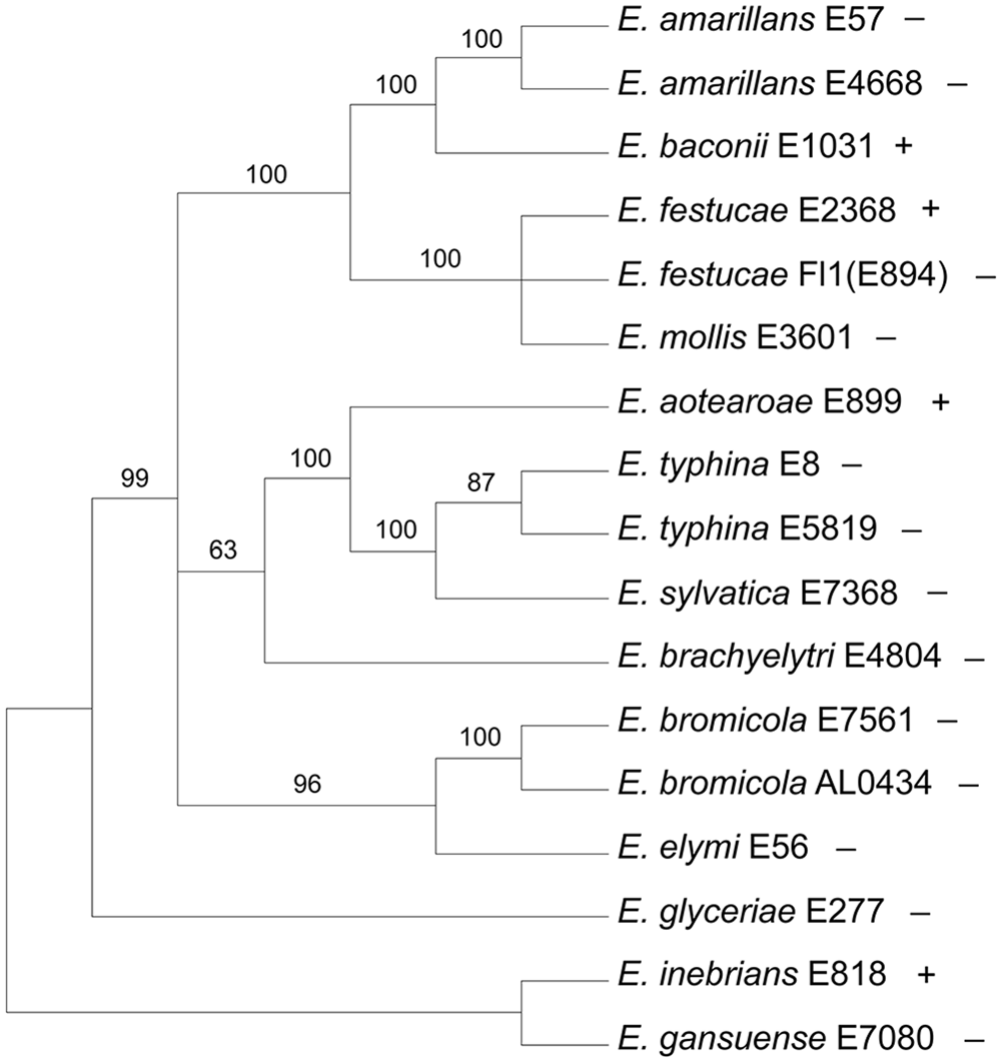
Rooted 50% majority rule maximum parsimony phylogenetic tree of the MCM7 gene DNA sequences (including introns). The *E*. *gansuensis* and *E*. *inebrians* sequences were designated as the outgroups for rooting the tree. The presence (+) or absence (−) of an antifungal protein gene in the genome sequence is indicated for each species in the tree. The numbers at the nodes are the bootstrap percentages based on 1,000 replications. The tree was based upon 2,810 total characters, of which 2,492 were constant, 117 variable characters were parsimony uninformative, and 201 variable characters were parsimony informative. Available NCBI accession numbers of the contigs containing the sequences are: *E*. *amarillans* E57, AFRF01000144; *E*. *amarillans* 4668, JFGZ01000314; *E*. *aoteraoae*, JFGX01000075; *E*. *baconii*, JFGY01000038; *E*. *brachyelytri*, AFRB01001052; *E*. *bromicola* ALO4262, LBNH01000152; *E*. *bromicola* ALO434, LBNI01000418; *E*. *elymi*, AMDJ01000289; *E*. *festucae* E2368, ADFL02000041; *E*. *festucae* Fl1, AFRX02000143; *E*. *gansuense*, AFRE01000016; *E*. *glyceriae*, AFRG01000147; *E*. *inebrians*, AMDK01000026; *E*. *sylvatica*, LCTT01000222; *E*. *typhina* E8, AMDI01000042; *E*. *typhina* E5819, AFSE01000068.

We previously reported that genes similar to the *E*. *festucae* antifungal protein gene were not found in most *Epichloë* spp. for which whole genome sequences were available, being present in only *E*. *festucae* 2368 and *E*. *inebrians* as well as the *E*. *festucae* RC isolate used in our study^17^. Since then genome sequences of additional *Epichloë* spp. have been reported^37^ revealing that similar antifungal protein genes are present in the more recently sequenced genomes of *E*. *baconii* and *E*. *aotearoae*. To illustrate the evolutionary relationships among the *Epichloë* spp. that possess an antifungal protein gene, a maximum parsimony phylogenetic analysis was conducted of the conserved MCM7 gene, including introns, of the 17 *Epichloë* isolates for which whole genome sequences are available (Fig. 8). The MCM7 gene encodes a subunit of the minichromosome maintenance complex^38^ and is frequently used in fungal phylogenies^39, 40^. The *E*. *inebrians* and *E*. *gansuensis* sequences were designated as outgroups for rooting the tree since they are considered the basal *Epichloë* spp.^41, 42^. The presence or absence of an antifungal protein gene in each species is indicated on the MCM7 phylogenetic tree. The patchy distribution of the presence of an antifungal protein gene throughout the *Epichloë* spp. suggests there have been numerous instances of gene loss.

**Figure 9:**
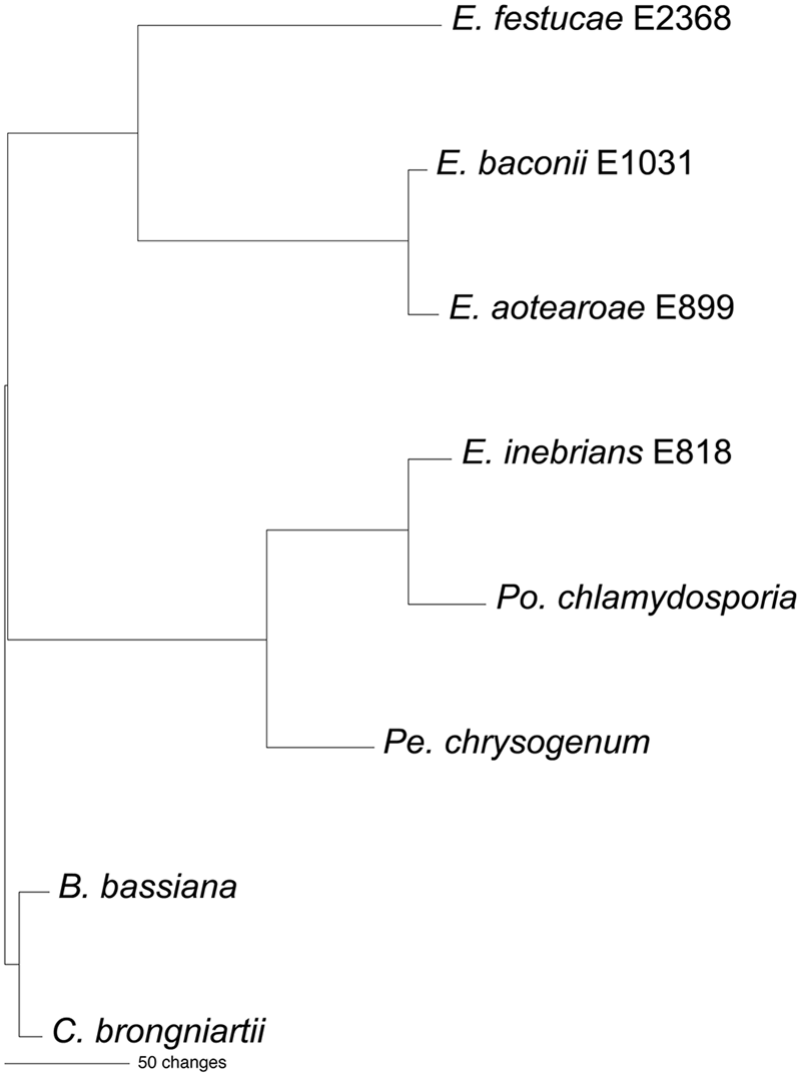
The single most parsimonious phylogenetic tree from an exhaustive search of antifungal protein gene DNA sequences (including introns). The *B*. *bassiana* and *C*. *brongniartii* sequences were designated as the outgroups for rooting the tree. The tree was based upon 435 total characters, of which 161 were constant, 55 variable characters were parsimony uninformative, and 219 variable characters were parsimony informative. NCBI accession numbers for the sequences are: *B*. *bassiana* ARSEF 2860, ADAH01000656; *C*. *brongniartii* RCEF 3172, AZHA01000055; *E*. *aotearoae* 1229, JFGX01000916.1; *E baconii* 200745, JFGY01002353.1; *E*. *festucae* E2368, ADFL02000476.1; *E*. *inebrians* E818, AMDK01000862.1; *Pe*. *chrysogenum*, U22944.2; *Po*. *chlamydosporia* 123, AOSW01001534.1.

A phylogenetic analysis of the antifungal protein gene DNA sequences, including introns, of the *Epichloë* spp. is shown in Fig. 9. A previous phylogenetic analysis of antifungal protein DNA coding sequences from Eurotiomycete and Sordariomycete fungi showed that the antifungal protein PAF from *Pe*. *chrysogenum* (Trichocomaceae) was more closely related to similar proteins from Sordariomycetes than those from other Eurotiomycetes^17^ and so is included in the phylogeny in Fig. 9. The coding sequence of the other similar antifungal protein from *Pe*. *chrysogenum*, PgAFP, is phylogenetically close to similar sequences from other Eurotiomycetes, as would be expected^17^, and is not included in Fig. 9. Also included is the sequence from *Pochonia chlamydosporia* (Clavicipitaceae), a parasite of nematode eggs^43^. The sequences from the insect pathogens *Beauveria bassiana* and *Cordyceps brongniartii* (Cordycipitaceae)^44, 45^ were designated as outgroups for rooting the tree. In this analysis the antifungal protein gene from *E*. *inebrians* was more closely related to those of *Po*. *chlamydosporia* and *Pe*. *chrysogenum* than to the other *Epichloë* spp. genes. The *E*. *inebrians* sequence is 86% and 72% identical to the *Po*. *chlamydosporia* and *Pe*. *chrysogenum* sequences, respectively, whereas it is only 58–59% identical to the other *Epichloë* sequences (data not shown). The sequence similarity of *E*. *inebrians* to the *Po*. *chlamydosporia* and *Pe*. *chrysogenum* sequences, throughout the coding sequence as well as in the intron sequences, is readily apparent in the sequence alignment (Supplementary Fig. S2).

**Figure 10:**
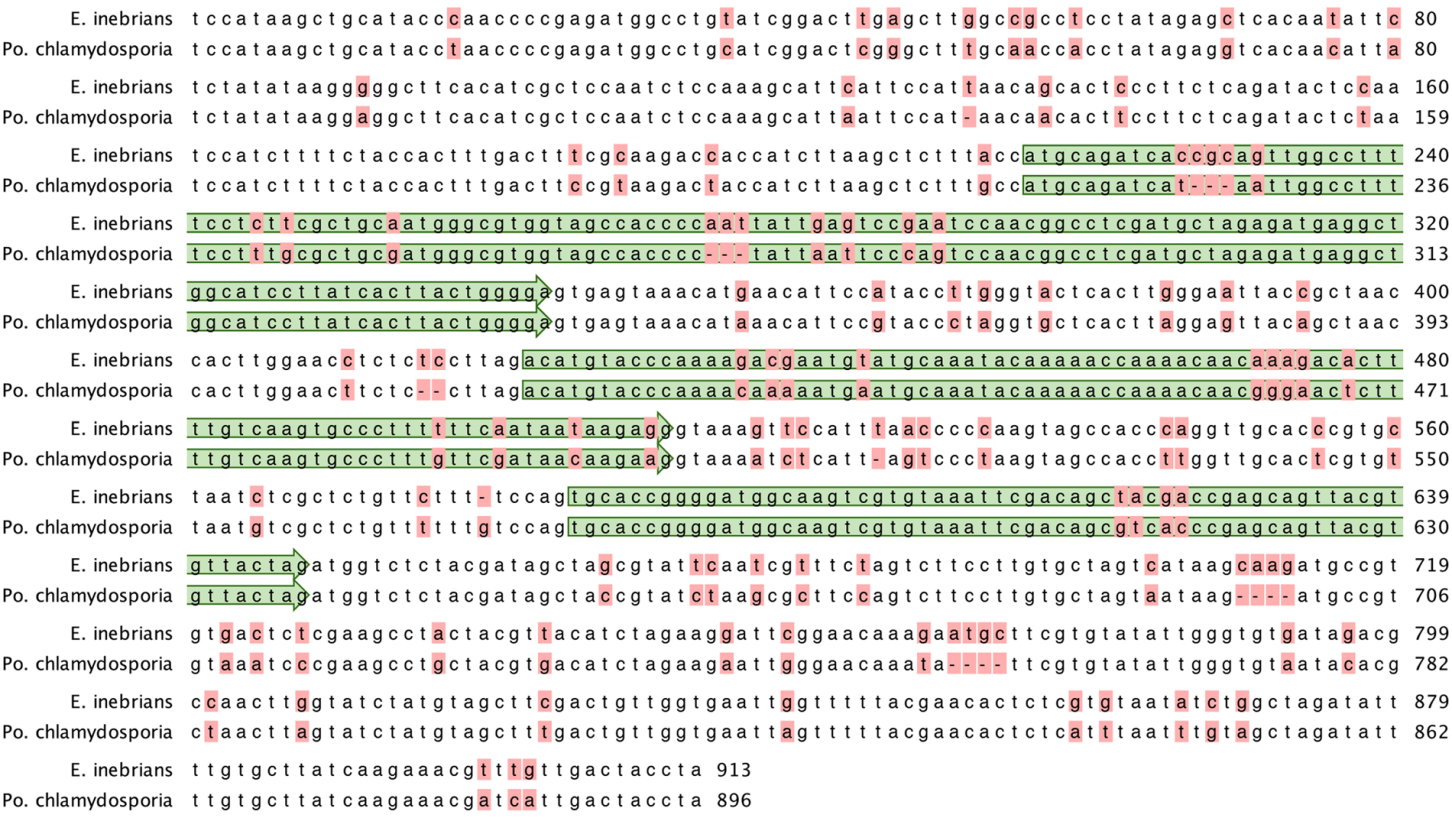
Similarity of the *E*. *inebrians* and *Po*. *chlamydosporia* antifungal protein DNA sequences extends through the upstream and downstream regions of the gene. The antifungal protein coding sequences are boxed in green. Differences between the two sequences are boxed in red.

The phylogenetic analysis of the antifungal protein gene suggests that the evolutionary history of the gene in *E*. *inebrians* may be different from that of the other *Epichloë* spp. and may have involved horizontal gene transfer. Horizontal gene transfer of a large genomic island containing the PAF sequence has been reported in *Penicillium* spp. isolated from food environments^46^. In reciprocal blastn searches of the NCBI fungal genome sequences, the *E*. *inebrians* and *Po*. *chlamydosporia* antifungal protein gene sequences were the best matches to each other. There was 88% identity over a span of 913 nucleotides, including 215 bases upstream of the initiation ATG codon and 266 bases downstream of the TAG stop codon (Fig. 10). Genome sequences for 2 isolates of *Po*. *chlamydosporia* are available at NCBI, but the antifungal protein gene is present in the genome of only one of the isolates. The *E*. *inebrians* and the *Po*. *chlamydosporia* antifungal protein genes appear to have a common origin that may have involved horizontal gene transfer.

### Genomic Context of the *E*. *festucae* Antifungal Protein Gene

**Figure 11:**
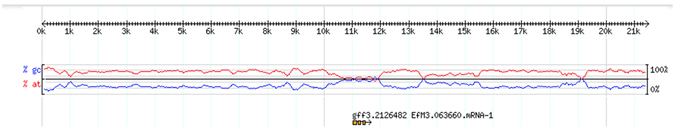
*Efe*-*afpA* is the only gene on a genome sequence contig consisting mainly of repeated sequences. Screenshot of *E*. *festucae* E2368 supercontig 268 from the *Epichloë festucae* Genome Project website^5^ (http://csbio-l.csr.uky.edu/ef2011/gbrowse/ef/).

Since the phylogenetic analyses described above suggest numerous instances of antifungal protein gene loss among the *Epichloë* spp., as well as the likelihood of a separate evolutionary history for the gene in *E*. *inebrians*, the genomic contexts of the antifungal protein genes in the four *Epichloë* spp. were compared. *Efe*-*afpA* is the lone gene, flanked by repeated sequences, on a 21504 bp genome sequence contig of *E*. *festucae* 2368 (Fig. 11)^5^ (http://csbiol.csr.uky.edu/ef2011/gbrowse/ef/?q=scaffold00268). The high A/T regions of the contig are the repeated sequences. Since *Efe*-*afpA* is the single gene on the contig it is not possible to determine if the gene order is syntenic with the similar genes in the other *Epichloë* spp. Similarly, the *E*. *aotearoae*, *E*. *baconii*, and *E*. *inebrians* antifungal protein genes are also located on small contigs of 5425, 1982, and 7529 bp, respectively.

## Discussion

Here we have reported the characterization of an *E*. *festucae* antifungal protein that is highly expressed in the symbiotic association with its grass host, strong creeping red fescue. The transcript for the protein had been previously reported as being the second most abundant fungal transcript in infected strong creeping red fescue^17^ and, based on the peptide sequencing reported here, the protein itself is abundant in the plant apoplast. The abundance of the antifungal protein in the host plant’s apoplast suggests it could come into contact with invading fungal pathogens and may therefore be a factor in the fungal disease resistance observed in *E*. *festucae*-infected strong creeping red fescue. This hypothesis is supported by the antifungal activity assays of the purified protein from the apoplastic proteins and the recombinant protein purified from the culture filtrate of the yeast *P*. *pastoris*. Viability assays of *S*. *homoeocarpa* after treatment with *Efe*-AfpA using SYTOX Green and Evans blue suggested that *Efe*-AfpA inhibited growth of the pathogen by causing damage to the plasma membrane. Similar results were found for AFP from *A*. *giganteus* ^47^.

Two versions of the *Efe*-AfpA protein were expressed in *P*. *pastoris*, the mature form and a version with a 6 amino acid N-terminal extension that was also detected among the apoplastic proteins. Both forms did have antifungal activity, but the mature form had considerably more activity. *Efe*-AfpA and the similar antifungal proteins from other fungal species are all secreted proteins with a signal sequence, a pro-sequence, and apparently an additional short amino acid sequence that is processed after secretion from the cell. Many proteins have pro-sequences, presumably for proper folding^48^. Mature PAF, lacking the pro-sequence, expressed in bacteria had no activity suggesting a requirement for the pro-sequence for proper folding^27^. However, when mature forms of *Efe*-AfpA, and the similar PAF, AFP, and NFAP proteins were expressed in *P*. *pastoris*, all lacking the pro-sequence, they could fold into active forms^32, 34, 35^. Apparently the eukaryotic cellular environment of *P*. *pastoris* supports proper folding of the proteins even in the absence of the pro-sequences.

To confirm the involvement of the *E*. *festucae* antifungal protein in the observed disease resistance *in planta*, a gene knockout was attempted. The gene knockout strategy was based on the homologous recombination strategy that has been successful for other *E*. *festucae* genes^49^ using hygromycin resistance as a selectable marker. However, no knockouts were recovered from screening over 200 hygromycin resistant transformants. This is likely attributable to the unusual genomic context of the antifungal protein in which the small gene is surrounded by repeated sequences. Homologous recombination in filamentous fungi typically requires long regions of homology^50^. Successful gene knockouts in *E*. *festucae* generally have 1.5 to 2.5 kb of flanking sequences surrounding the selectable marker and result in 1–25% of transformants being knockouts^49^. For the *E*. *festucae* antifungal protein, only 598 and 634 bp of upstream and downstream unique sequences, respectively, were available for producing the knockout construct. The short regions of homology are likely the reason no gene knockouts were obtained. In the future, another approach will be required for generating an antifungal protein gene knockout.

An *E*. *festucae* E437 isolate from the grass *Festuca pulchella* Schrad. (soft fescue) was reported to produce a small (<3500 kDa), thermostable antifungal compound whose expression was controlled by a transcription factor designated VibA^51, 52^. That antifungal compound differs from the *Efe*-AfpA described here, since *Efe*-AfpA was retained by a 3 kDa molecular weight cut off (MWCO) centrifugal filter unit, whereas the antifungal compound from *E*. *festucae* E437 was not retained by 3.5 kDa MWCO dialysis tubing. In the future it will be interesting to determine if either of these two *E*. *festucae* isolates is capable of producing both antifungal compounds. The genome sequence of *E*. *festucae* E437 is not currently available.

Most *Epichloë* spp. for which whole genome sequences are available do not have a gene similar to *Efe*-*afpA*. Phylogenetic analysis suggested that the similar genes in *E*. *baconii* and *E*. *aotearoae* are evolutionarily related to *Efe*-*afpA*. However, the similar gene in *E*. *inebrians* was more evolutionarily related to genes in *Po*. *chlamydosporia* and *Pe*. *chrysogenum*, suggesting it may have originated from horizontal gene transfer, possibly from *Po*. *chlamydosporia*. Horizontal gene transfer is considered an important factor in fungal genome evolution^53^ and extensive horizontal gene transfer was reported between Magnaporthales and *Colletotrichum* ^54^. There have been few reports of horizontal gene transfer in *Epichloë* spp. An *Epichloë* toxin gene similar to the bacterial insect toxin genes *makes caterpillars floppy* (*mcf1* and *mcf2*) was proposed to have originated from horizontal gene transfer from a bacterial donor since at that time, among the hundreds of fungal genome sequences available, only *Epichloe* spp. had such a gene^41^. Since then more fungal genomes have been sequenced and *mcf*-like genes are found in a few additional fungal genomes. However, the most similar to the *Epichloë* spp. *mcf*-like gene are in the distantly related phylum Basidiomycota, suggesting that the direct donor to the *Epichloë* spp. may have been another fungal species, rather than a bacterial species. An extensive computational analysis of the *E*. *festucae* genome did not identify any likely horizontally transferred genes^55^.

In summary, the abundant secreted protein *Efe*-AfpA was shown to have activity against an important fungal turfgrass pathogen, *S*. *homoeocarpa*, the causal agent of dollar spot disease. *Efe*-AfpA may therefore be an important factor in the disease resistance observed in this symbiotic association between *E*. *festucae* and its host grass, strong creeping red fescue. By protecting its grass host from fungal pathogens the fungal endophyte *E*. *festucae* is sustaining and protecting its own living environment.

## Methods

### Plant and Fungal Materials

The strong creeping red fescue (*Festuca rubra* subsp. *rubra*) plants used in this study, S1139E- and S1139 Rose City (S1139RC), have been described previously^56^. The endophyte-infected plant S1139RC was generated by inoculating an isolated tiller of the uninfected strong creeping red fescue plant S1139E- with the Rose City isolate of *E*. *festucae*, which was isolated from an unrelated endophyte-infected strong creeping red fescue. The plants S1139E- and S1139RC are thus the same plant genotype. Plants were clonally propagated and maintained in the greenhouse. These plants have been stably maintained in the greenhouse for over 15 years. The endophyte status of the plants was confirmed microscopically^57^ before initiation of this study. The *E*. *festucae* Rose City isolate (*E*. *festucae* RC) was isolated from the endophyte-infected S1139RC plant by plating surface-sterilized leaf sheath tissue on potato dextrose agar (Difco Laboratories, Detroit, MI, USA).

### Isolation of Apoplastic Proteins

Isolation of apoplastic proteins was conducted by using a modification of the method previously described by Moy *et al*.^58^. Two g of leaves and leaf sheaths were cut into 2-cm segments, rinsed, and vacuum infiltrated for 30 min with 20 mL of 100 mM Tris-HCl, pH 8.0, 50 mM dithiothreitol, 10 mM ascorbic acid, and 1 mM Pefabloc SC protease inhibitor (Sigma-Aldrich, St. Louis, MO, USA). The tissue was washed with water three times, blotted dry, and collected in a 10-cc syringe. The syringe was placed in a 30 mL Corex glass tube and centrifuged at 2,000 × g for 10 min at 4 °C. The apoplastic fluid was collected in a microfuge tube. Protein concentrations were determined by using the Bio-Rad Protein Assay Dye Reagent Concentrate (Bio-Rad Laboratories, Hercules, CA, USA).

For sodium dodecyl sulfate-polyacrylamide gel electrophoresis (SDS-PAGE) analysis, protein samples were mixed with 5x SDS sample buffer (5:1, v/v)^59^, then boiled for 5 min and subjected to electrophoresis in 16% polyacrylamide gels. Gels were stained with Coomassie Blue to visualize protein bands. Gels were destained, and then dried using a modified protocol from Moghaddam and Reinton^60^ as described previously^61^. Gels were soaked in a solution of 50% (v/v) methanol and 20% (v/v) polyethylene glycol 400 (PEG-400). Cellophane sheets, first soaked in water, were used to sandwich the gels in a gel-drying frame. Gels were dried at room temperature overnight.

### Peptide Sequencing

For peptide sequencing, the target band was excised from the SDS-PAGE gel and washed twice with 50% acetonitrile in water. Sequence analysis of peptides in the band from the endophyte-infected apoplastic proteins was performed at the Harvard Mass Spectrometry and Proteomics Resource Laboratory (Cambridge, MA, USA) by microcapillary reverse-phase HPLC nano-electrospray tandem mass spectrometry (LC/MS/MS) on a Thermo LTQ-Orbitrap mass spectrometer. Peptide sequence analysis of the antifungal protein partially purified from the apoplastic proteins was performed at the Biological Mass Spectrometry Facility at Rutgers University (Piscataway, NJ, USA) by LC/MS/MS on a Thermo Q Exactive HF mass spectrometer with a Dionex U-3000 rapid separation nano LC system.

### Partial Purification of *Efe*-AfpA From the Apoplastic Proteins

Apoplastic fluid from 4 g of leaves was collected as described above and mixed with an equal volume of 100 mM NaPO_4_, 3.0 M (NH_4_)_2_SO_4_ (pH 7.0). The sample was then applied to a 1-mL HiTrap phenyl HP column (GE Healthcare Life Sciences, Piscataway, NJ) and the column washed with 5 mL of 50 mM NaPO_4_, 1.5 M (NH_4_)_2_SO_4_ (pH 7.0) to remove unbound proteins. *Efe*-AfpA was eluted with five mL of 50 mM NaPO_4_, 1 M (NH_4_)_2_SO_4_ (pH 7.0). One mL aliquot fractions were collected. The buffer was exchanged to 50 mM NaPO_4_ (pH 7.0) and the protein was concentrated by using Amicon Ultra-0.5 mL 3 kDa centrifugal filters (EMD Millipore, Billerica, MA, USA).

### Production of the *E*. *festucae* Antifungal Protein in *Pichia pastoris*

*Efe*-*afpA* was expressed in *Pichia pastoris* by using the EasySelect *Pichia* Expression kit (Invitrogen by Life Technologies, Carlsbad, CA, USA). Two expression vectors were constructed based on the longer form of *Efe*-AfpA recovered from the apoplastic proteins beginning with AET, designated *Efe*-AfpA-2, and the most abundant mature form of *Efe*-AfpA from the apoplastic proteins beginning with ITY, designated *Efe*-AfpA-3. The *Efe*-*afpA* coding sequences were cloned into the Xho 1 and Not 1 sites of expression vector pPICZα A, a vector designed to target the recombinant protein for secretion from the yeast cells by cloning downstream of the *Saccharomyces cerevisiae* α-factor mating signal sequence. The *Efe*-*afpA* coding sequences were amplified by PCR with oligonucleotides that introduced a Xho 1 site and the Kex2 site at the 5′ ends and a Not 1 site at the 3′ end. The Ste13 cleavage sites were not included since the STE13 protease has been reported to inefficiently cleave the resulting protein^34, 62^. The oligonucleotides used for PCR amplification of the coding sequences are presented in Table 1.

**Table 1:** Sequences of oligonucleotide primers used in this study.

Forward primer, 5′–3′	Reverse primer, 5′–3′
Cloning into pPICZα A	
*Efe*-*afpA*-2	
GTATCTCTCGAGAAAAGAGCGGAGACTGGCATTCTGA	AAGCTGGCGGGCCGCCTAATGACACGTGACAGCTC
*Efe*-*afpA*-3	
GTATCTCTCGAGAAAAGAATCACGTATGAAGGAACATGTT	AAGCTGGCGGGCCGCCTAATGACACGTGACAGCTC
Deletion of propeptide and Kex2 sites of the α-factor signal sequence	
*Efe*-*afpA*-3	
ATCACGTATGAAGGAACATGTTC	AGCTAATGCGGAGGATGC
Sequencing recombinant plasmids	
5′ AOX1 primer	
GACTGGTTCCAATTGACAAGC	

For both constructs, the amplification reactions were carried out in a GeneAmp 9700 thermo-cycler (Applied Biosystems, Foster City, CA, USA). The 100 μL reactions contained 2X Phusion High-Fidelity PCR Master Mix with HF Buffer (Thermo Fisher Scientific, Waltham, MA, USA), 40 pmol of each oligonucleotide, and 80 pg plasmid DNA as template. The PCR reaction conditions were: an initial denaturation step at 94 °C for 30 s, followed by 30 cycles of 10 s denaturation at 98 °C, 30 s annealing at 59 °C, and 30 s extension at 72 °C. An additional final 5 min extension at 72 °C was performed. The PCR products were purified by using 1.8X Agencourt AMPure XP (Beckman Coulter, Brea, CA, USA).

The purified *Efe*-*afpA* PCR products were digested with FastDigest Xho 1 and Not 1 restriction enzymes (Thermo Fisher Scientific). The PCR products were first digested with 1 μL Not 1 at 37 °C for 30 min, then 0.5 μL of Xho 1 was added to the reaction and the incubation continued for 4 min. The samples were then heated at 80 °C for 5 min to inactivate the enzymes. Since there is a Xho 1 site within the *Efe*-*afpA* coding sequence, the Xho 1 digest was short to facilitate recovery of partially digested PCR products. The expression vector pPICZα A was also digested with FastDigest Xho 1 and Not 1 restriction enzymes (Thermo Fisher Scientific) followed by treatment with shrimp alkaline phosphatase (Affymetrix, Santa Clara, CA, USA) to prevent vector relegation. The digested PCR products and vector were purified by using 1.8 X Agencourt AMPure XP. Ligation of the digested PCR products (22 ng) and pPICZα A (200 ng) was with T4 DNA ligase (Invitrogen, Carlsbad, CA, USA) overnight at room temperature.

Two μL of the ligation products were used to transform 20 μL *E*. *coli* electroporation-competent TOP10F’ cells. The transformed cells were incubated in low-salt LB medium for 1 hour at 37 °C with shaking, followed by overnight growth of cells on low salt LB medium supplemented with 25 μg mL^−1^ zeocin. Transformed bacterial colonies were screened for recombinant plasmids containing the *Efe*-*afpA* coding sequence inserts by using PCR as described above. The PCR products were visualized on a 2% TBE agarose gel. Plasmids from selected positive bacterial colonies were isolated and sequenced (Genewiz, Inc., South Plainfield, NJ, USA) using the 5ʹ AOX1 sequencing primer (Table 1).

Protein sequencing (described above) of the recombinant *Efe*-AfpA-3 revealed that the protein was not efficiently processed at the Kex2 site since a prominent peptide recovered was RITYEGTCSR. Therefore, another construct for *Efe*-*afpA*-*3* was generated by deleting the α-factor propeptide sequence including the Kex2 site in the plasmid, such that the primary α-factor signal sequence cleavage site was adjacent to the start of the mature *Efe*-*afpA*-*3* sequence. The Q5 Site-Directed Mutagenesis Kit (New England BioLabs Inc., Ipswich, MA, USA) was used to modify the plasmid containing the mature form of *Efe*-*afpA*-*3* by deleting the plasmid propeptide α-factor coding sequence of 66 amino acids, including the Kex2 signal cleavage site, downstream of the experimentally determined 19 amino acid α-factor signal sequence^63^ (Table 1).

The pPICZα A empty vector and the recombinant plasmids containing the *Efe*-*afpA* coding sequences were linearized by FastDigest Mss 1 (Thermo Fisher Scientific) and then purified by using 1.8X Agencourt AMPure XP. The purified linearized plasmids were transformed into *P*. *pastoris* KM71H (*arg4 aox1*::*ARG4*) cells by electroporation. The *P*. *pastoris* cells were prepared for electroporation as described in the EasySelect Pichia Expression Kit manual. Selected transformant *P*. *pastoris* clones were grown and protein expression induced by methanol addition for 5 days as described in the kit manual. The culture filtrate was then collected by centrifuging at 3000 × g at 25 °C for 5 min.

*Efe*-AfpA-2 and *Efe*-AfpA-3 were partially purified from the *P*. *pastoris* culture filtrate by first removing proteins larger than 30 kDa by passing through Amicon Ultra-15 molecular weight cutoff 30 kDa centrifugal filters (Sigma-Aldrich) at 3000 × g at 4 °C. Proteins in the flow-through were then concentrated by using Amicon Ultra-15 molecular weight cutoff 10 kDa centrifugal filters at 3000 × g at 4 °C. Proteins from the culture filtrate of the pPICZα A empty vector control *P*. *pastoris* transformant were prepared in the same way.

### Antifungal Activity Assays

*S*. *homoeocarpa* was maintained on potato dextrose agar (PDA) containing 50 μg mL^−1^ kanamycin. Since *S*. *homoeocarpa* does not produce spores, all antifungal activity assays were carried out on mycelium harvested from the PDA plates. A plate assay was used to qualitatively determine the antifungal activity of *Efe*-AfpA purified from apoplastic fluid against *S*. *homoeocarpa*. A thin layer of *S*. *homoeocarpa* fungus (1 cm^2^) was cut and placed at the center of a PDA plus kanamycin plate. The plate was incubated at room temperature for one day. Thirty microliters of the partially purified antifungal protein (1 μg μL^−1^) and 30 μL of 50 mM NaPO_4_ were added to opposite sides of the plate. The plate was incubated at room temperature. A zone of no growth of the pathogen, indicative of antifungal activity, was apparent after 1 d.

A 24-well plate assay was used to qualitatively determine the antifungal activity of *Efe*-AfpA expressed in *P*. *pastoris* on *S*. *homoeocarpa*. Prior to use in the antifungal assays, the partially purified proteins from the *P*. *pastoris* culture filtrates were filter sterilized by passing through a 0.2 μm syringe filter (Corning Incorporated, Corning, NY, USA). A mycelial plug of fungus (1.5 cm × 1.5 cm) was cut from a 7 d culture of the fungus on a PDA plate and ground in 3 mL potato dextrose broth (PDB) by using a sterile glass dounce homogenizer. The pathogen suspension was mixed with *Efe*-AfpA-2, *Efe*-AfpA-3, or the empty vector transformant proteins purified from the *P*. *pastoris* culture filtrate in 2:1, 1:1, and 1:2 volume to volume (v:v) ratios, in a total of 60 μL. The amount of purified proteins in the mixtures was 30 μg, 45 μg, and 60 μg, respectively. The pathogen/protein mixtures were spread onto PDA in a 24-well plate and incubated at room temperature.

A 35-mm petri dish plate assay was used to quantitatively determine the antifungal activity of *Efe*-Afp-2, *Efe*-Afp-3, or the empty vector transformant proteins against *S*. *homoeocarpa*. Three replicates each of *Efe*-AfpA-2, *Efe*-AfpA-3, or empty vector transformant proteins purified from the *P*. *pastoris* culture filtrate were mixed with PDA in final concentrations of 2, 50, 100, 200, and 400 μg mL^−1^. Mycelial plugs of *S*. *homoeocarpa* (0.5 cm diameter) were cut from a 7 d culture of the fungus on a PDA plate and placed at the center of the 35-mm petri dishes containing the PDA-antifungal protein mixtures and incubated at room temperature. The colony diameters (cm) were measured daily, and mean values were used to quantitatively determine inhibitory activity.

### Microscopy

The SYTOX Green uptake assay was modified from a method described previously^47^. A mycelial plug of *S*. *homoeocarpa* (0.2 cm × 0.2 cm) was cut from a 7 d culture of the fungus on a PDA plate and ground in 3 mL potato dextrose broth (PDB) by using a sterile glass dounce homogenizer. Two hundred μL of the fungal suspension were cultivated in a 96-well cell culture plate at room temperature for 2 d. Seventeen μg of *Efe*-AfpA-3 or empty vector transformant proteins purified from the *P*. *pastoris* culture filtrate were added and incubated for 2 h. SYTOX Green was added to a final concentration of 12 μM. Fluorescence was examined and photographed with a Carl Zeiss Axioskop microscope with the filter set at an excitation wavelength of 450 to 490 nm and an emission wavelength at 515 to 565 nm.

The Evans blue staining assay was modified from Shehata *et al*.^64^. Five hundred μL of PDA plus 50 μg mL^−1^ kanamycin was spread on a sterilized glass slide in a petri dish and allowed to solidify. A fragment of *S*. *homoeocarpa* from a 7 d culture of the fungus on a PDA plate was applied to the center on the agar. Thirty-five micrograms of *Efe*-AfpA-3 or empty vector transformant proteins purified from the *P*. *pastoris* culture filtrate was added to the side of the *S*. *homoeocarpa* fungal fragment on each slide. Slides were incubated at room temperature for 1 d. They were then stained with a solution of 1% Evans blue for 15 min, followed by washing with water for 15 min, and examined and photographed with a Carl Zeiss Axioskop microscope.

### Phylogenetic Analysis

The MCM7 (gene model EfM3.060700) and antifungal protein (gene model EfM3.063660) gene sequences were obtained from the Genome Project at the University of Kentucky website^5, 31^ (http://www.endophyte.uky.edu/). The Clustal-X program was used to align the sequences^65^. The alignments generated by Clustal-X were modified manually to minimize gaps. The phylogenetic analysis was performed with the PAUP* program, version 4.0b10 for Macintosh. The MCM7 phylogenetic analysis was done by using the maximum parsimony full heuristic search option set to random sequence addition, tree-bisection-reconnection (TBR) branch swapping, and Multrees on, with 1000 bootstrap replications. Gaps were treated as missing data. The antifungal protein gene and protein phylogenetic trees were done by using an exhaustive maximum parsimony analysis, which returned a single most parsimonious tree in both cases.

### Data Availability

All data generated or analysed during this study are included in this published article (and its Supplementary information files).

## Electronic supplementary material

Supplementary Information

Dataset 1
