# Supplementary material for: The Epichloë festucae antifungal protein has activity against the plant pathogen Sclerotinia homoeocarpa, the causal agent of dollar spot disease
Source: Sci Rep. 2017 Jul 17;7:5643. doi: 10.1038/s41598-017-06068-4 (PMC5514056; doi:10.1038/s41598-017-06068-4)
Supplement: Supplementary file 1 — Supplementary Information [file 41598_2017_6068_MOESM1_ESM.pdf]

Supplementary Information

The *Epichloë festucae* antifungal protein has activity against the plant pathogen *Sclerotinia homoeocarpa*, the causal agent of dollar spot disease

Zipeng Tian[1], Ruying Wang[1], Karen V. Ambrose[1, 2], Bruce B. Clarke[1], Faith C. Belanger[1*]

[1] Department of Plant Biology, Rutgers University, New Brunswick, New Jersey 08901,

[2] Current address: Indigo Agriculture, Charlestown, Massachusetts 02129

Supplementary Fig. S1. The single most parsimonious phylogenetic tree from an exhaustive search of the antifungal protein amino acid sequences shown in Fig. 2. The tree is midpoint rooted. The tree was based upon 101 total characters, of which 26 were constant, 56 variable characters were parsimony uninformative, and 19 variable characters were parsimony informative. NCBI accession numbers for the sequences are *Efe*-AfpA from *E. festucae*, SRR493691.12929; PAF from *P. chrysogenum*, U22944.2; PgAFP from *P. chrysogenum*, D0EXD3.1; NFAP from *N. fischeri* , AM983570, and AFP from *A. giganteus*, accession X60771.

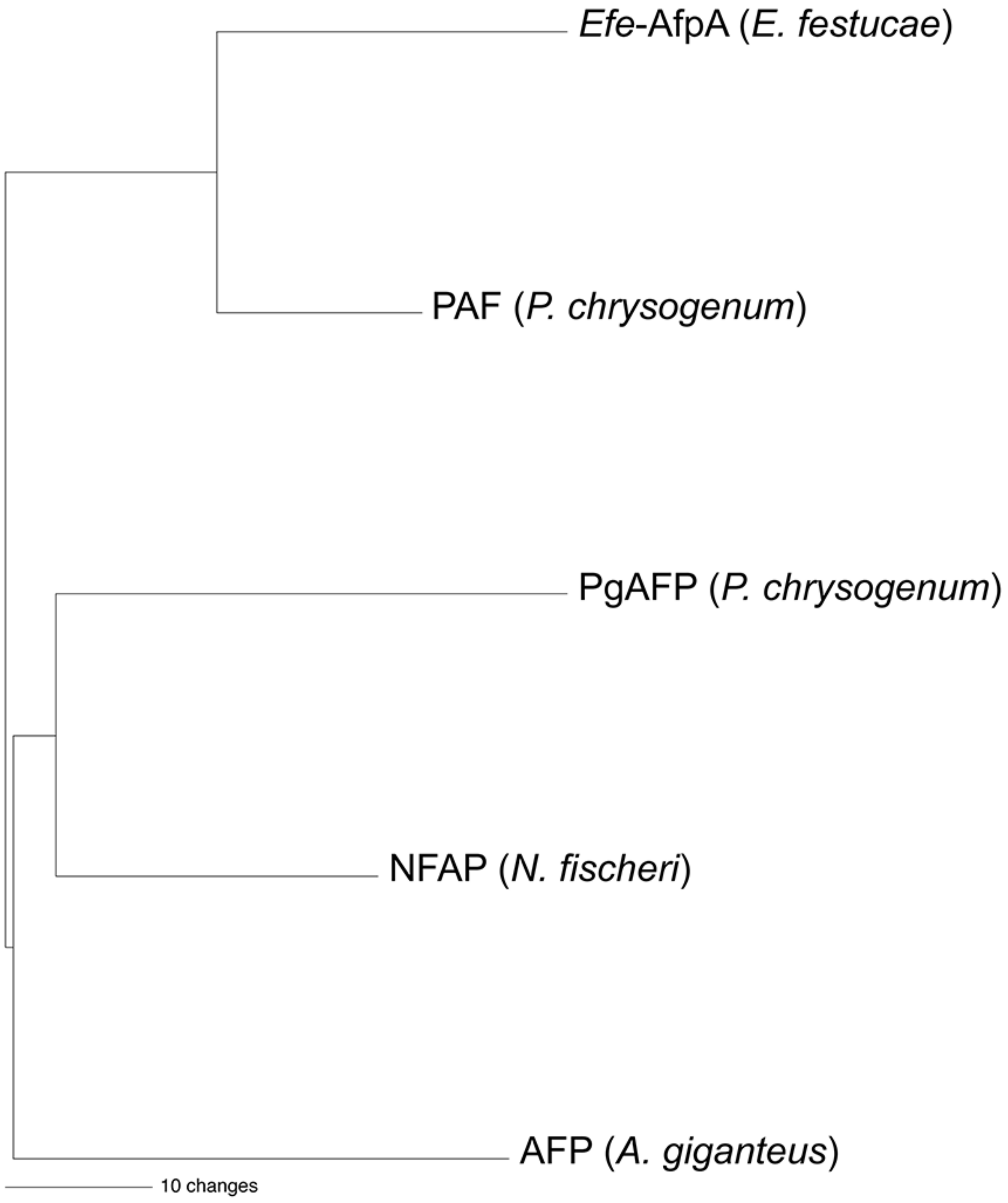
Efe-AfpA (*E. festucae*)
PAF (*P. chrysogenum*)
PgAFP (*P. chrysogenum*)
NFAP (*N. fischeri*)
AFP (*A. giganteus*)
10 changes

Supplementary Fig. S2. DNA sequence alignment of the antifungal protein gene sequences used for the phylogentic analysis presented in Figure 8. The coding sequences are in upper case and the intron sequences are in lower case. Asterisks indicate regions of identity.

```
E. festucae               ATGCAAATCACCGTGGTCGCGGTTTTCCTCCTCTCTGCAATGGGCGGAGT
E. baconii                ATGCAAATCACCGTAGTCGCGGTTTTCCTCCTCTCTGCAATAGGCGGAGC
E. aotearoae              ATGCAAATCACCGTAGTCGCGGTTTTCCTCCTCTCTGCAATAGGCGGAGC
E. inebrians              ATGCAGATCACCGCAGTTGGCCTTTTCCTCTTCGCTGCAATGGGCGTGGT
Po. chlamydosporia        ATGCAGATCA---TAATTGGCCTTTTCCTTTGCGCTGCGATGGGCGTGGT
Pe. chrysogenum           ATGCAAATCACCACAGTTGCCCTTTTTCTCTTCGCTGCAATGGGCGGGGT
B. bassiana               ATGCAGATTATTTCCATCGCACTCTCTCTCCTCGCTGCGACCGGCGCCGT
C. brongniartii           ATGCAAATTATTTCCATCGCCCTCTTTCTCCTCACTGCGACCGGCGCCGT
                          ***** ** *      * *   * *  **   * **** *  ****  *

E. festucae               AGCCACACC---CATCAACTCCAGAATCAATCCTGTTGATGCCAGGGCGG
E. baconii                AGCCACACC---CATTAACTCCAGAATCAATCATGTTGATGCCAAAGCGG
E. aotearoae              AGCCACACC---CATTAACTCCGGAATCAATCATGTTGATGCCAAAGCGG
E. inebrians              AGCCACCCCAATTATTGAGTCCGAATCCAACGGCCTCGATGCTAGAGATG
Po. chlamydosporia        AGCCACCCC---TATTAATTCCCAGTCCAACGGCCTCGATGCTAGAGATG
Pe. chrysogenum           AGCCACCCC---CATTGAGTCTGTATCAAACGACCTCGATGCCAGGGCTG
B. bassiana               CGCCGCCGC------------CACCCCTGAGCAGTTTGAGGCCAGAGATG
C. brongniartii           CGCCGCCGC------------CACCCCTGAGCAGTTTGAGGCCAGAGATG
                           *** *  *                    *     * ** ** *  *  *

E. festucae               AGACTGGCATTCTGATCACGTATGAAGGA gtgagttgat-tttgtgtcac
E. baconii                AGACTGGCGTTCTGATCACGTATACAGGA gtgagtcaat-tttgtgttac
E. aotearoae              AGACTGGCGTTCTGATCACGTATATAGGA gtgagtcaat-tttgtgttac
E. inebrians              AGGCTGGCATCCTTATCACTTACTGGGGA gtgagtaaacatgaacattcc
Po. chlamydosporia        AGGCTGGCATCCTTATCACTTACTGGGGA gtgagtaaacataaacattcc
Pe. chrysogenum           AGGCCGGTGTCCTGGCCAAATACACCGGA gtgagtaaacatcaatatccc
B. bassiana               GGGCCGGCGCCATGATCAAGTACCACGGA gtaagcaag-----ataccgc
C. brongniartii           GGGCCAGCGCCATGATCAAGTACCACGGA gtgagcaag-----aaaccgc
                           * *  *     *   **  **    *** ** **               *

E. festucae               aatacc-----cgattactctgagtattcaa-taacca-cggagtcccctc
E. baconii                aatacc-----ctattactctaattcttcaa-taacca-cggaatcccctc
E. aotearoae              aatacc-----ctattactctaattactcaa-taacca-cggaatcccctc
E. inebrians              -ataccttgggtactcacttgggaattaccgctaaccacttggaacctctc
Po. chlamydosporia        -gtaccctaggtgctcacttaggagttacagctaaccacttggaacttctc
Pe. chrysogenum           -ataccttaagtactcacttgggaatcgcgactaacggttcgggaccacaa
B. bassiana               -caatcc---atgttcactacggag-caaagctaacca-tccactcaactt
C. brongniartii           -caatcc---atgttcactacggag-caaagctaacca-tcgaatcacgtt
                             * *        * ***             ****         *

E. festucae               tataaag ACATGTTCTAGAGCCAAAAACGAATGCAAGTACAAAAATCAGA
E. baconii                tataaag ACATGTACTAGAGACAAAAACGAATGCAAGTACAAAAATCAGA
E. aotearoae              tataaag ACATGTACTAGAGCCAAAAACGAATGCAAGTACAAAAATCAGA
E. inebrians              tccttag ACATGTACCCAAAAGACGAATGTATGCAAATACAAAAACCAAA
Po. chlamydosporia        --cttag ACATGTACCCAAAACAAAAATGAATGCAAATACAAAAACCAAA
Pe. chrysogenum           --ctcag AAATGCACCAAATCTAAGAACGAATGTAAATACAAGAACGATG
B. bassiana               --caaag ATTTGCACCAAGGCCAAGAACGAATGCAAGTTCAAGGGCCAGA
C. brongniartii           --caaag ATTTGCACCAAGGCTAAGAACGAATGCAAGTTCAAGGGCCAGA
                               ** *  **  *       *  ** * *** ** * ***     *

E. festucae               ACAACAAGGACACGTTCGTCAAGTGCCCGTCGTTCGCAAACAAGAAG gta
E. baconii                ACAATAAGAACACGTTCGTCAAGTGCCCGTCGTTTGCAAACAAGAAG gta
E. aotearoae              ACAATAAGGATACGTTCGTCAAGTGCCCTTCGTTTGCAAACAAGAAG gta
E. inebrians              ACAACAAAGACACTTTTGTCAAGTGCCCTTTTTTCAATAATAAGAGG gta
```

```
Po. chlamydosporia       ACAACGGGAACTCTTTTGTCAAGTGCCCTTTGTTCGATAACAAGAAG gta
Pe. chrysogenum          CTGGAAAGGACACTTTTATCAAGTGCCCCAAGTTTGATAACAAGAAG gta
B. bassiana              ATGGCAGGGATACCTTTGTCAAGTGCCCCAACTTTGCCAACAAGAGG gta
C. brongniartii          ACGGCAAGGATACCTTTGTCAAGTGCCCCAACTTTGCCAACAAGAGG gta
                                  *  * **  **********    **    ** **** * **

E. festucae              aaggttctttttttgccccccttttgaggct--gcctatttccaagcttacg
E. baconii               aaggttctttttttaccccccttttcaggct--gcttatttccaagcttacg
E. aotearoae             aaggttctttttttaccccccttttcaggct--gcttatttctaagcttacg
E. inebrians             aagttccatttaacccccaagtagccacccaggttgcacccgtgctaatc
Po. chlamydosporia       aaatctcatt-agtccctaagtagccaccttggttgcactcgtgttaatg
Pe. chrysogenum          gaatatcaat-cattcggaagtagccatctgaatcgatttcgtgctaatc
B. bassiana              cg----tatttctccctcgacaagccgct--aattgtgtagatgctgact
C. brongniartii          cg----tatttctccctcgacaagctgct--aattgtttagatgctgact
                                  *     *                           * * *

E. festucae2368          tctgattaatccaccgttacag TGCACCAAAGACAACGCCAAGTGTAGCT
E. baconii               tctgtttaatccaccgttacag TGCACCAAAGACAAAGCCAAGTGTACCT
E. aotearoae             cgtttttaattcatcgttacag TGCACCAGAGACGGCGCCAAGTGTACCT
E. inebrians             tcgctctgttc---ttttccag TGCACCGGGGATGGCAAGTCGTGTAAAT
Po. chlamydosporia       tcgctctgttt--tttgtccag TGCACCGGGGATGGCAAGTCGTGTAAAT
Pe. chrysogenum          tcgctct------tttttccag TGCACCAAGGATAATAACAAATGTACCG
B. bassiana              gc-----------tttctttag TGCACCAAGGACTACAACGAATGTTCAT
C. brongniartii          gc-----------tttctttag TGCACCAAGGACCACAACGAATGTTCGT
                                          *  ** ******   **          ***

E. festucae              TTGACAGTTATTCTCGAGCTGTCACGTGTCATTAG
E. baconii               TTGACAGTGTTACTCGAGGTGTCACGTGTGATTAG
E. aotearoae             TTGACAGTGTTTCTCGAGGTGTCACGTGTGATTAG
E. inebrians             TCGACAGCTACGACCGAGCAGTTACGTGTTACTAG
Po. chlamydosporia       TCGACAGCGTCACCCGAGCAGTTACGTGTTACTAG
Pe. chrysogenum          TCGACACCTACAACAACGCTGTCGATTGTGACTAG
B. bassiana              ATGACAGCGTCAGCCGAGCTGTCGTGTGCCACTAG
C. brongniartii          ACGACAGCGTCACCCGAGCTGTCGTGTGCCACTAG
                           ****           *  **    **  * ***
```
